# Identification of genetic variants of the IL‐22 gene in association with an altered risk of COPD susceptibility

**DOI:** 10.1111/crj.13517

**Published:** 2022-07-09

**Authors:** Yan Wang, Qipeng Zhou, Lingzhu Chen, Lian Dong, Mingmei Xiong, Xiaohui Xie, Li Zhao, Jingyi Xu, Zeguang Zheng, Jian Wang, Wenju Lu

**Affiliations:** ^1^ State Key Laboratory of Respiratory Diseases, Guangzhou Institute of Respiratory Health, The First Affiliated Hospital Guangzhou Medical University Guangzhou China

**Keywords:** COPD, IL22, promoter region activity, SNP

## Abstract

The incidence of chronic obstructive pulmonary disease (COPD) is related to the interaction between environmental exposure and genetic factors. Far more than 15% of smokers eventually develop COPD. In addition to smoking, genetic susceptibility may be another factor in the development of COPD. IL‐22 and its receptors are increased in human and experimental COPD and contribute to pathogenesis. Here, we conducted a case–control study to evaluate the association between IL‐22 tag‐single nucleotide polymorphisms (SNPs) and COPD risk. Four tag‐SNPs (rs2227478, rs2227481, rs2227484 and rs2227485) were identified according to linkage disequilibrium (LD) analysis in 30 healthy controls. A total of 513 COPD cases and 504 controls were recruited to perform an association study between these four tag‐SNPs and COPD risk. We found that the “C” allele of rs2227478T>C and the “T” allele of rs2227481C>T were obviously related to decreased COPD susceptibility. Genetic model analysis showed that rs2227478T>C and rs2227481C>T were significantly associated with a decreased risk of COPD under dominant models after adjusting for the above factors. In the recessive model, rs2227485T>C was obviously associated with decreased COPD risk. Our data showed that only rs2227485T>C was associated with a decreased COPD risk after Bonferroni correction. The eQTL analysis showed that rs2227485T>C was significantly associated with IL‐22 expression. The pGL4‐rs2227485‐C gene reporter had a higher promoter activity than pGL4‐rs2227485‐T. In our study, rs2227485T>C, located in the promoter region of IL‐22, was associated with a decreased risk of COPD and increased IL‐22 promoter activity, suggesting that this variant might modulate COPD susceptibility.

## Graphical abstract

The rs2227485T>C, located in the promoter region of IL‐22, was associated with a decreased risk of chronic obstructive pulmonary disease (COPD) and increased IL‐22 promoter activity, suggesting that this variant might modulate COPD susceptibility.

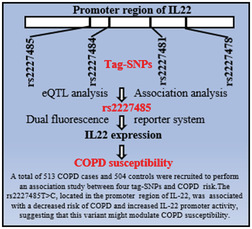

## 1 BACKGROUND

Chronic obstructive pulmonary disease (COPD) is characterised by chronic bronchitis and/or emphysema with airflow obstruction. It has been reported that the global prevalence of COPD is approximately 11.4%,^1^ and the prevalence of COPD in people over 40 years old is 13.6% in China.^2^ COPD is one of the top three causes of death worldwide.^3^ The incidence of COPD is related to the interaction of environmental exposure and genetic factors. Tobacco smoking is the leading risk factor for environmental exposure. Far more than 15% of smokers eventually develop COPD.^4^ Family history contributes to 18.6% of the population‐attributable risk of COPD. Patients with a family history of COPD have more severe symptoms, more frequent exacerbation and worse quality of life than the control group.^5^ In addition, the two main phenotypes of COPD showing independent family aggregation may be related to different genetic factors.^6^

Single nucleotide polymorphisms (SNPs) mainly refer to DNA sequence polymorphisms caused by the mutation of a single nucleotide variation in the genome, including transformations, transversions, deletions and insertions. SNPs are the most common heritable variations in humans. A large number of diseases are affected by SNPs, such as type 1 diabetes mellitus and Graves' disease and Hashimoto's autoimmune thyroiditis.^7^ Many studies have proven that SNPs in genes, such as FAM13A, CHRNA5/3 and HTR4, are closely associated with susceptibility to COPD.^8^, ^9^, ^10^, ^11^

The transcript of interleukin (IL)‐22 was first found in mouse thymus T cells, and the base sequences encoding IL‐22 and IL‐10 genes were found to be homologous.^12^ IL‐22 can mediate cellular inflammatory responses. The encoded protein functions in antimicrobial defence at mucosal surfaces and in tissue repair. A variety of immune cells secrete IL‐22, such as CD4, CD8 and γδ T lymphocytes, natural killer cells and innate lymphoid cells (ILCs).^13^ This protein also has proinflammatory properties and plays a role in the pathogenesis of COPD.^14^ Increased IL‐22 levels and IL‐22 + cells have been demonstrated in the blood, sputum and lung biopsies of COPD patients.^15^ The role of IL‐22 in lung antimicrobial defence and the impact of COPD on this defence pathway have been reported.^16^, ^17^ Most studies have focused on the protein function of interleukin 22. At present, there are few reports of associations between SNPs of IL‐22 and COPD. Whether these SNPs affect the expression of the IL‐22 gene and thus affect the development of COPD remains to be explored. Here, we aimed to identify functional SNPs located on IL‐22 and its upstream and downstream 3 kb regions associated with COPD.

## 2 METHODS

### 2.1 Study populations

The human samples were all from the Guangzhou Institute of Respiratory Disease (GIRD) COPD Biobank, which has been described previously.^18^, ^19^ COPD was diagnosed based on the criteria of the National Heart Lung and Blood Institute (NHLBI)/WHO Global initiative for chronic obstructive lung disease (GOLD).^20^ COPD was defined as FEV1/FVC less than 70% after inhalation of bronchodilation with some chronic airway symptoms, including chronic cough, dyspnoea, sputum production or wheezing. The controls who had normal spirometry results were randomly selected from the Health Examination Center of the same hospital during the same time period that the patients were recruited. A standardised epidemiological questionnaire was designed to collect demographic and personal data, and information, such as personal details (age, sex, ethnicity and residential region) and smoking history, was included. A total of 1017 participants (sex matched) were recruited after excluding patients with asthma, lung cancer, pulmonary fibrosis, bronchiectasis and other respiratory diseases. All subjects were unrelated to the Chinese Han population. Peripheral blood and genomic DNA were also extracted from all samples. All patients signed the consent form. The study passed the ethical approval of the First Affiliated Hospital of Guangzhou Medical University (GZMC2009‐08‐1336).

### 2.2 Lung function

The details of pulmonary function measurement have been described previously.^19^ Briefly, pulmonary ventilation tests and bronchodilator tests were performed at the First Affiliated Hospital of Guangzhou Medical University. The forced vital capacity (FVC), forced expiratory volume in the first second (FEV1) before and after bronchodilator, forced expiratory volume in the first second (FEV1%) and forced ventilator percentage in the first second (FEV1/FVC%) were recorded.

### 2.3 Selection and genotyping of tag‐SNPs

SNPs satisfying the following two conditions were selected. First, those located in the sequence from 3 kb upstream to 3 kb downstream of the IL‐22 gene were selected from the dbSNP database (www.ncbi.nlm.nih.gov/snp, GRCh37p13, 29/11/2014). Second, SNPs with a minor allele frequency (MAF) > 5% were selected. Finally, 11 SNPs (supporting information Table S1) were identified by this strategy. These SNPs were genotyped by polymerase chain reaction (PCR) and Sanger sequencing in 30 randomly selected healthy control subjects. The tag‐SNPs among them were further identified using Haploview 4.2 software (https://www.broadinstitute.org/haploview/haploview) based on linkage disequilibrium (LD) analysis (*r* ^2^ > 0.8). The detailed methods have been described previously.^21^

The genomic DNA from the 1017 participants was extracted using the QIAamp DNA Blood Mini Kit (QIAGEN Co. Ltd., Germany). We genotyped the tag‐SNPs with an efficient multiple gene region enrichment/next‐generation sequencing‐based assay by Genesky Biotechnologies, Inc. (Shanghai, China).

### 2.4 Plasmid construction

The methods of plasmid construction were previously reported.^22^ Briefly, the rs2227485T>C‐C and rs2227485T>C‐T allele reporter constructs were prepared by amplifying a 2 kb 5′ promoter of human IL‐22, which was used with the forward primer: 5′‐ CGGGGTACCTGTATCGTTGTACCCGTTGA‐3′ and reverse primer: 5′‐GGAAGATCTATGAAGGTGCGGTTGGTGAT‐3′. The pGL4‐Basic vector was digested with KpnI and BglII restriction enzymes (Thermo, USA), and then, the purified PCR products were ligated into the pGL4‐Basic vector upstream of the firefly luciferase gene at the T4 DNA ligase to produce pGL4‐rs2227485T>C‐C and pGL4‐rs2227485T>C‐T reporter plasmids. All constructs were sequenced to ensure the correct sequence, orientation and integrity of each insert. The function of the cloned fragments on downstream gene expression was detected by firefly luciferase activity with Renilla luciferase as an internal standard.

### 2.5 Plasmid transfection and measurement of luciferase activity

Lipofectamine® 3000 DNA Transfection Reagent (Thermo. USA) was used to perform the plasmid transfection according to the instructions in the 293 T cell line. Luciferase activity was measured by the Dμal‐Glo® Luciferase Assay System (Promega, USA) according to the manufacturer's instructions.

### 2.6 eQTL analysis

SNPexp v1.2 (http://app3.titan.uio.no/biotools/help.php?app=snpexp) integrates the HapMap project database and the GENEVAR database. We performed correlation analysis between genotypes and target gene expression using PLINK, a tool for genome‐wide association analysis. We used SNPexp v1.2 to analyse whether the SNPs rs2227478, rs2227481, rs2227484 and rs2227485 were related to IL‐22 gene expression in the CHB population.

### 2.7 Statistical analysis

All data are expressed as X ± S. Allele frequencies and genotypes of the four tag‐SNP loci in the case group and the control group were statistically analysed for Hardy–Weinberg equilibrium, odds ratio (OR) value and 95% confidence interval (CI) using SPSS 22.0 and STATA MP13 software. A two‐tailed *P* < 0.05 was considered statistically significant. In addition, Pearson's *χ* ^2^ and Fisher's exact tests were used to calculate the allele frequencies of cases and controls, and MAF in controls was defined as baseline. After adjusting for age, sex and smoking status, ORs and 95% CIs were calculated using unconditional logistic regression analysis. The relationship between the selected SNPs and the risk of COPD was calculated using genotypic model analysis (codominant, dominant, recessive, overdominant and log additive) by the website software programme SNPStats.^23^ Student's *t* test was used to compare the differences in quantitative data if the data followed a normal distribution; otherwise, the *χ* ^2^ test was used. Comparison of dual fluorescence reporter gene activity was tested by Student's t test.

## 3 RESULTS

### 3.1 Demographic characteristics of COPD cases and controls

A total of 513 (432 males) COPD cases and 504 (414 males) controls were included in this study. As shown in Table 1, there was no significant difference in sex distribution (84.21% male vs. 82.14% male, *P =* 0.789) or body mass index (BMI) (20.90 ± 3.52 vs. 22.80 ± 2.25 kg/m2, *P <* 0.05) between the two groups. The ages in the case group were significantly older than those in the control group (68.59 ± 7.95 vs. 56.00 ± 9.83 years, *P <* 0.05). COPD patients had a much higher number of pack‐years than control participants (42.00 ± 24.73 vs. 22.49 ± 17.83, *P <* 0.05).

**TABLE 1:** Data of the study population

Characteristics	Cases	Controls	*P*‐value
Subject (*n*)	513	504	
Age (years)	68.59 ± 7.95	56.00 ± 9.83	<0.05
Male (%)	84.21	82.14	0.789
BMI	20.90 ± 3.52	22.80 ± 2.25	<0.05
Pack‐year	42.00 ± 24.73	22.49 ± 17.83	<0.05
FEV_1_ (L)	1.04 ± 0.49	1.91 ± 0.61	<0.05
FEV_1_/pred (%)	41 ± 18	70 ± 23	<0.05
FVC (L)	2.27 ± 0.69	2.78 ± 0.52	<0.05
FEV_1_/FVC (%)	46 ± 18	67 ± 15	<0.05

*Note*: *P* < 0.05 indicates statistical significance.

Abbreviations: BMI, body mass index; FEV, forced expiratory volume; FVC, forced vital capacity.

### 3.2 Identification of tag‐SNPs in the IL‐22 gene

Supporting information Figure S1 shows that four LD blocks were found by performing LD analysis (*r* ^2^ > 0.8). We selected one SNP from each LD site as a tag‐SNP site. The position of the genome, MAF and functional area is shown in supporting information Table S2. All four SNPs were located in the 5′ near zone of the gene.

### 3.3 Association analysis of IL‐22 tag‐SNPs with COPD risk

As shown in Table 2, the calling rates of SNPs were all over 95%. The “T” allele of rs2227484C>T had no effects on COPD susceptibility in our selected population [OR (95% CI) = 0.88 (0.65–1.19), *p =* 0.419]. The “T” allele of rs2227478T>C [OR (95% CI) = 0.75 (0.59–0.95), *p =* 0.019] and the “C” allele of rs2227481C>T [OR (95% CI) = 0.62 (0.44–0.88), *p =* 0.007] were obviously related to increased COPD susceptibility. It is worth noting that the “C” allele of rs2227485T>C had a trend of decreased COPD susceptibility in the recruited population [OR (95% CI) = 0.85 (0.71–1.02), *p =* 0.083].

**TABLE 2:** Allele frequency distribution of tag‐SNPs in IL‐22 gene

SNP	Allele (major/minor)	Call rate	MAF		*P*‐value	OR(95%CI)	HWE^a^
			Cases (*n* = 503)	Controls (*n* = 514)			
rs2227478	T/C	96.17%	0.14	0.18	0.019*	0.75 (0.59–0.95)	0.29
rs2227481	C/T	98.03%	0.06	0.09	0.007*	0.62 (0.44–0.88)	0.16
rs2227484	C/T	98.72%	0.09	0.1	0.419	0.88 (0.65–1.19)	0.8
rs2227485	T/C	95.28%	0.45	0.49	0.083	0.85 (0.71–1.02)	0.28

*Note*: *P < 0.05 indicates statistical significance.

Abbreviations: CI, confidence interval; HWE, Hardy–Weinberg equilibrium; MAF, minor allele frequency; OR, odds ratio; SNP, single nucleotide polymorphism.

^a^ HWE, Hardy–Weinberg equilibrium among the control subjects.

Table 3 shows the associations between the four tag‐SNPs and COPD risk under various genetic models. Compared with the common wild‐type homozygous genotype “TT” of rs2227485T>C, the “CC” genotype had a trend of decreased COPD risk after adjusting for correction factors, including sex, age, BMI and pack‐years, in the codominant genetic model [OR (95% CI): 0.66 (0.42–1.03), *p =* 0.0048]. rs2227478T>C and rs2227481C>T were significantly associated with a decreased risk of COPD under dominant models after adjusting for the above factors [OR (95% CI): 0.67 (0.48–0.95), *p =* 0.025; 0.58 (0.37–0.92), *p =* 0.02, respectively]. In the recessive model, rs2227485T>C was obviously associated with decreased COPD risk [OR (95% CI): 0.56 (0.38–0.82), *p =* 0.0032]. Our data showed that only rs2227485T>C was associated with a decreased COPD risk after Bonferroni correction [OR (95% CI): 0.56 (0.38–0.82), Bonferroni test *p =* 0.0128].

**TABLE 3:** Genetic models analysis of tag‐SNPs

SNP	Genotype	Cases	Controls	Codominant		Dominant		Recessive	
				OR (95% CI)	*P*‐value	OR (95% CI)	*P*‐value	OR (95% CI)	*P*‐value
rs2227478	T/T	359 (73.7%)	331 (67.4%)	1	0.081	1	0.025*	1	0.52
	T/C	116 (23.8%)	140 (28.5%)	0.67 (0.47–0.97)		0.67 (0.48–0.95)			
	C/C	12 (2.5%)	20 (4.1%)	0.67 (0.27–1.67)				0.74 (0.30–1.84)	
rs2227481	C/C	447 (89%)	410 (82.8%)	1	0.066	1	0.02*	1	0.92
	C/T	54 (10.8%)	84 (17%)	0.58 (0.37–0.92)		0.58 (0.37–0.92)			
	T/T	1 (0.2%)	1 (0.2%)	0.77 (0.03–21.76)				0.84 (0.03–23.46)	
rs2227484	C/C	424 (83.6%)	405 (81.5%)	1	0.7	1	0.44	1	0.63
	C/T	78 (15.4%)	87 (17.5%)	0.86 (0.56–1.32)		0.85 (0.56–1.28)			
	T/T	5 (1%)	5 (1%)	0.67 (0.14–3.15)				0.68 (0.14–3.23)	
rs2227485	T/T	142 (29.3%)	134 (27.7%)	1	0.0048	1	0.75	1	0.0032*
	C/T	252 (52%)	230 (47.5%)	1.31 (0.90–1.89)		1.06 (0.75–1.49)			
	C/C	91 (18.8%)	120 (24.8%)	0.66 (0.42–1.03)				0.56 (0.38–0.82)	

*Note*: Codominant: W/W vs W/V vs V/V; dominant: W/V, V/V vs W/W; recessive: V/V vs W/W, W/V; *P*‐value: adjusted by gender, age, smoking index.

Abbreviations: CI, confidence interval; OR, odds ratio; SNP, single nucleotide polymorphism.

* *P* < 0.05 indicates statistical significance.

### 3.4 Stratification and eQTL analysis of IL‐22 tag‐SNPs

As mentioned above, smoking is an important factor in COPD. Whether the polymorphism of the IL‐22 gene interacts with tobacco smoke exposure, thus affecting the risk of COPD, is still unknown. We performed a stratified analysis according to smoking status in the recruited population. As shown in Table 4, we observed a statistically significant difference in the distribution of heterozygous and wild‐type genotypes (*p =* 0.015) for rs2227481C>T in the smoker group compared with the control group. Compared with the heterozygous genotype “CC” of rs2227481C>T, the wild‐type genotype “CT” was associated with a decreased the risk of COPD [OR (95% CI) = 0.513 (0.298–0.880), *p =* 0.015]. It is worth noting that compared with the wild‐type genotype “TT” of rs2227478T>C, the heterozygous genotype “TC” had a trend of being a protective factor for COPD [OR (95% CI) = 0.649 (0.419–1.01), *p =* 0.053]. The genotype “CC” of rs2227485T>C was associated with a lower risk of COPD than the wild‐type genotype “TT” [OR (95% CI) = 0.591 (0.348–1.00), *p =* 0.050]. In the nonsmoker group, no significant differences were found in the distribution of genotypes at the four tag‐SNPs. All of the above *p*‐values were calculated by adjusting for correction factors, including sex, age, BMI and pack‐years.

**TABLE 4:** Smoking stratified analysis of tag‐SNPs

SNP	Genotype	Smokers				Nonsmokers			
		Case	Control	*P*‐value	OR (95% CI)	Case	Control	*P*‐value	OR (95%CI)
rs2227478	C/C	10	16	0.593	0.734 (0.237–2.278)	2	2	0.659	0.558 (0.042–7.456)
	T/C	82	106	0.053	0.649(0.419–1.01)	31	29	0.342	0.691(0.323–1.479)
	T/T	266	236		1	87	78		1
rs2227481	T/T	1	1	0.584	2.432 (0.101–58.499)	0	0	‐	‐
	C/T	41	70	0.015*	0.513 (0.298–0.880)	12	12	0.639	1.294 (0.441–3.796)
	C/C	328	290		1	111	98		1
rs2227484	T/T	4	4	0.487	0.418 (0.036–4.880)	1	0		1
	C/T	54	57	0.997	0.999 (0.590–1.692)	22	24	0.078	0.469 (0.202–1.090)
	C/C	314	300		1	102	88		1
rs2227485	C/C	64	93	0.050	0.591 (0.348–1.00)	26	22	0.750	0.849 (0.312–2.312)
	C/T	181	159	0.476	1.172 (0.757–1.816)	65	57	0.355	1.479 (0.645–3.392)
	T/T	110	102		1	30	27		1

*Note*: *P < 0.05 indicates statistical significance. *P*‐value: adjusted by gender, age, smoking status.

Abbreviations: CI, confidence interval; OR odds ratio; SNP, single nucleotide polymorphism.

We used eQTL to analyse whether these four SNPs were associated with gene expression. Table 5 shows that rs2227485T>C was significantly associated with IL‐22 expression after Bonferroni correction (*β =* 0.02931, *p =* 0.01808).

**TABLE 5:** eQTL analysis of tag‐SNPs

CHR	SNP	Position	Allele	*β*	*P‐*value
12	rs2227485	66 933 980	T/C	0.02931	0.01808*
12	rs2227484	66 934 196	C/T	−0.01451	0.4174
12	rs2227481	66 934 608	C/T	−0.03916	0.05617
12	rs2227478	66 934 889	T/C	−0.03277	0.03996

Abbreviations: CHR: chromosome; β, the regression coefficient; SNP, single nucleotide polymorphism.

* *P* < 0.05 indicates statistical significance after Bonferroni correction.

### 3.5 Effects of rs2227485T>C polymorphisms on IL‐22 gene reporter activity

**FIGURE 1:**
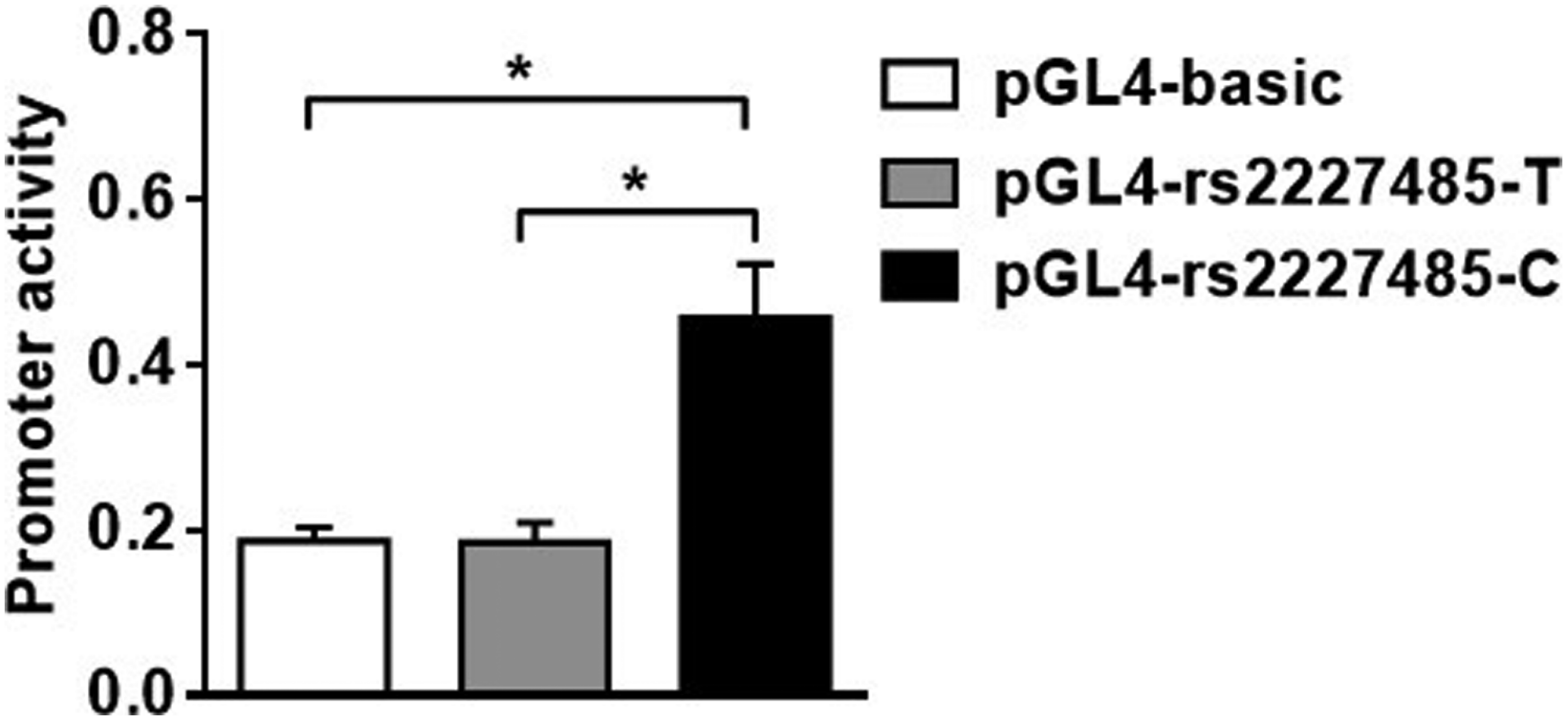
Dual fluorescence report detection of rs2227485 expression vector. pGL4‐basic: Empty vector; pGL4‐rs2227485‐C: Expression vector with allele C of rs2227485;pGL4‐rs2227485‐T: Expression vector with allele T of rs2227485; ordinate: Relative promoter activity of the expression vector; *indicates P < 0.05, which is regarded as a significant difference, *N* = 6, *P < 0.05.

rs2227485T>C had significant differences in genotype distribution in the recessive genetic model compared with the control. This site is located in the promoter region of the IL‐22 gene, 485 bp upstream of the transcription initiation point; thus, it may play a role in regulating the transcription of the IL‐22 gene. Therefore, we used a dual fluorescence reporter system to analyse whether the two alleles at this site can regulate the activity of the IL‐22 gene promoter. Figure 1 shows no significant difference in promoter activity between pGL4‐rs2227485‐T and pGL4‐basic (0.18 ± 0.06 vs. 0.19 ± 0.04, *n* = 6, *p =* 0.94). pGL4‐rs2227485‐C showed significantly higher promoter activity than pGL4‐rs2227485‐T (0.46 ± 0.16 vs. 0.18 ± 0.06, *n* = 6, *p =* 0.003). The above results suggest that rs2227485T>C can affect the activity of the IL‐22 gene promoter region.

## 4 DISCUSSION

To our knowledge, we are the first to perform an association study between tag‐SNPs of IL‐22 and COPD. In this study, we found that the “C” allele of rs2227478T>C and the “T” allele of rs2227481C>T located in the promoter region of the IL‐22 gene were associated with a decreased risk of COPD. In the recessive genetic model, the homozygous genotype of rs2227485T>C showed a higher protective effect on the risk of COPD than the heterozygous genotype. The three SNPs above may interact with cigarette smoking, thus participating in COPD development. Moreover, gene reporter activity detection indicated that allele “C” of rs2227485T>C had higher transcriptional activity than allele T and therefore increased IL‐22 gene expression.

Studies have demonstrated that IL‐22 contributes to COPD pathogenesis. The IL‐22‐IL‐22R signalling pathway is widely involved in innate and adaptive immune responses and plays an important role in regulating the host defence response and tissue inflammatory response and maintaining tissue homeostasis.^24^ Damage/repair imbalances are involved in the development of COPD.^25^ Increasing exogenous IL‐22 can promote the proliferation, survival and repair of epithelial cells and protect tissues by inducing the expression of bcl‐2, bcl‐x and Mcl‐1.^26^ Moreover, Malcolm R. Starkey et al found that IL‐22 promoted CS‐induced pulmonary neutrophilic inflammation, airway remodelling and lung function impairment. However, they also found that inhibiting IL‐22 can increase or decrease the risk of exacerbations due to its central role in pathogen clearance.^14^ Therefore, additional information, such as the relationships between IL‐22 and genetic factors in COPD, must be obtained prior to utilising therapeutic approaches targeting IL‐22 signalling. Recently, researchers performed a series of genome‐wide association studies of COPD and lung function.^27^, ^28^ They identified many new loci and described them in association with either COPD or population‐based lung function. These variants strongly predict COPD in independent populations. However, the four SNPs of IL‐22 did not appear in their list. One possible explanation is that the races in our study were different.

SNPs of IL‐22 have been proven to be associated with many diseases, including childhood cerebral malaria, liver cirrhosis and cancer risk.^29^, ^30^, ^31^ Marquet S et al. showed that individuals carrying the aggravating T allele of rs2227473 produced significantly more IL‐22 than those without this allele, which suggested that IL‐22 is involved in the pathogenesis of CM.^29^ Gao et al. revealed that the SNPs rs2227491 and rs1026788 of IL‐22 were associated with chronic hepatitis B virus infection progression.^30^ No studies have focused on the SNPs of IL‐22 affecting COPD risk. Our study first revealed that rs2227478T>C and rs2227481C>T located in the promoter area of IL‐22 were obviously related to decreased COPD susceptibility. rs2227478T>C was shown to have protective effects on autoimmune thyroid disease.^32^ Aljarba et al. found that rs2227481C>T located on IL‐22 could contribute to protection against P. falciparum malaria.^33^ However, some studies have shown that rs2227485T>C in IL‐22 might be associated with an increased risk of papillary thyroid cancer and gastric mucosa‐associated lymphoid tissue lymphoma.^34^ We demonstrated that rs2227485T>C in IL‐22 has a protective role in COPD. All these data indicate the functions of SNPs in varying diseases.

As mentioned, cigarette smoking is known to be an important factor in COPD. Many surveys have confirmed that SNPs could affect COPD risk through a response to smoking.^35^, ^36^ Whether the four tag‐SNPs affect the risk of COPD by interacting with cigarette smoking is still unknown. The stratified analysis on cigarette smoking indicated that rs2227481C>T and rs2227485T>C might contribute to COPD risk through interaction with cigarette smoking. Combining the results of the allele analysis and the genetic model analysis, we observed that the “C” alleles of rs2227478T>C and rs2227485T>C were protective factors for the development of COPD, which was consistent with the stratified analysis of cigarette smoking. Some functional SNPs have been identified as contributors to COPD susceptibility, such as FAM13A, HHIP and CHRNA 3/5.^8^, ^9^, ^37^, ^38^ Tang et al. demonstrated that the SNPs in CXCL10 could affect CXCL10 promoter activity and thereby contribute to CXCL10 expression.^39^ Based on the above studies, we further performed eQTL analysis to explore the relationship between the four tag‐SNPs analysed in our study and IL‐22 gene expression. We found that rs2227485T>C could be related to IL‐22 gene expression. In addition, the results of gene reporter activity reinforced the conclusion above. There are limitations to this study. First, a larger population is needed for verification of our findings, since the *p*‐value of the stratified analysis of rs2227485T>C in smoking did not reach statistical significance. Second, we only tested the allele function of rs2227485T>C, and we did not detect the mRNA and protein levels of IL‐22. The relationship between the IL‐22 expression level and the SNPs was still unclear. Further studies are warranted to elucidate how genotypes affect susceptibility. Third, COPD is a multifactorial and heterogeneous disease.^40^ Many genes may participate in the aetiology of COPD, and every single gene only partially contributes to each case of COPD. In addition, we did not include data for individuals with impaired spirometry (preserved ratio with impaired spirometry [PRISm]), as those data have to be analysed separately from data for individuals with FEV1% predicted> = 80%. We also did not analyse the isoforms in the eQTL analysis. Most importantly, we did not perform population stratification for factors such as XY sex and principal components of genetic ancestry, which can cause false associations in disease studies.

In summary, we first identified rs2227478T>C and rs2227481C>T as being associated with a decreased COPD risk. In addition, our study indicated that rs2227485T>C, a functional SNP located in the promoter area of IL‐22, partially participated in decreasing COPD risk by promoting IL‐22 gene promoter region activity. These findings might provide novel insight for exploring the pathogenesis of COPD and for developing new treatment strategies.

## CONFLICT OF INTEREST

The authors declare no financial or commercial conflict of interest.

## ETHICS STATEMENT

The project was approved by the institutional review boards of Guangzhou Medical University (Ethics Committee of The First Affiliated Hospital: GZMC2009‐08‐1336). All participants signed the consent form.

## AUTHOR CONTRIBUTIONS

Study design: Y.W. and W.J.L.; data collection: Y.W., Q.P.Z., L.Z.C., Z.Z.G., L.D, M.M.X., and X.H.X.; data quality control and analysis: L.Z., M.M.X., and J.Y.X.; statistical support: J.W.; experiments: L.Z.C., X.H.X., and Q.P.Z.; and manuscript writing: Y.W.. All authors revised the manuscript.

## Supporting information


**Supplementary Figure S1.** LD analysis of SNPs in IL‐22 gene: The tag‐SNPs among them were further identified using Haploview 4.2 software based on linkage disequilibrium (LD) analysis (r2 > 0.8).

**Supplementary Table S1.** Data of 11 SNPs located in from upstream to downstream 3 kb of IL22
**Supplementary Table S2.** Data of four tag‐SNPs

## Data Availability

The datasets used and/or analysed during the current study are available from the corresponding author on reasonable request.
