# Supplementary figures and images for: Identification of genetic variants of the IL‐22 gene in association with an altered risk of COPD susceptibility
Source: Clin Respir J. 2022 Jul 9;16(8):537–45. doi: 10.1111/crj.13517 (PMC9376143; doi:10.1111/crj.13517)

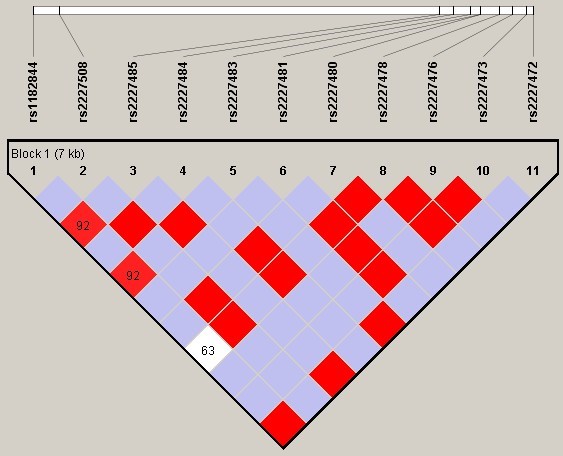

Supplement: Supplementary file 1 — Supplementary Figure S1. LD analysis of SNPs in IL‐22 gene: The tag‐SNPs among them were further identified using Haploview 4.2 software based on linkage disequilibrium (LD) analysis (r2 > 0.8). [file CRJ-16-537-s001.jpg]
