# Supplementary material for: Identification of genetic variants of the IL‐22 gene in association with an altered risk of COPD susceptibility
Source: Clin Respir J. 2022 Jul 9;16(8):537–45. doi: 10.1111/crj.13517 (PMC9376143; doi:10.1111/crj.13517)
Supplement: Supplementary file 2 — Supplementary Table S1. Data of 11 SNPs located in from upstream to downstream 3 kb of IL22 Supplementary Table S2. Data of four tag‐SNPs [file CRJ-16-537-s002.docx]

| **Supplement table1.** Data of 11 SNPs located in from upstream to downstream 3kb of IL22 | | | | | |
| --- | --- | --- | --- | --- | --- |
| Gene | dbSNP | Position | Allele | MAF | Area of Function |
| IL-22 | rs2227472 | chr12:68255353 | A/G | 0.4835 | 5' near gene |
|  | rs2227473 | chr12:68255258 | A/G | 0.1997 | 5' near gene |
|  | rs2227476 | chr12:68255036 | A/T | 0.1832 | 5' near gene |
|  | rs2227478 | chr12:68254842 | C/T | 0.3347 | 5' near gene |
|  | rs2227480 | chr12:68254562 | C/T | 0.1267 | 5' near gene |
|  | rs2227481 | chr12:68254561 | C/T | 0.1758 | 5' near gene |
|  | rs2227483 | chr12:68254396 | A/T | 0.4986 | 5' near gene |
|  | rs2227484 | chr12:68254149 | C/T | 0.1272 | 5' near gene |
|  | rs2227485 | chr12:68253933 | C/T | 0.4986 | 5' near gene |
|  | rs2227508 | chr12:68248148 | A/T | 0.1309 | 3' near gene |
|  | rs1182844 | chr12:68247752 | A/T | 0.3251 | 3' near gene |

**Abbreviation:** dbSNP, database of SNP; MAF: Minimum Allele Frequency;

| **Supplement table2.** Data of four tag-SNPs | | | | | |
| --- | --- | --- | --- | --- | --- |
| Gene | dbSNP | Position | Allele | MAF | Function |
| IL-22 | rs2227478 | chr12:68254842 | T/C | 0.3347 | 5' near gene |
|  | rs2227481 | chr12:68254561 | C/T | 0.1758 | 5' near gene |
|  | rs2227484 | chr12:68254149 | C/T | 0.1272 | 5' near gene |
|  | rs2227485 | chr12:68253933 | T/C | 0.4986 | 5' near gene |

**Abbreviation:** dbSNP, database of SNP; MAF: Minimum Allele Frequency
